# Novel insights into microbial DMSP/DMS cycling: from surface to deep ocean

**DOI:** 10.1128/mbio.01207-26

**Published:** 2026-06-09

**Authors:** Yunhui Zhang, Yanfen Zheng, Zihua Guo, Dong Liu, Xiao-Hua Zhang

**Affiliations:** 1 Frontiers Science Center for Deep Ocean Multispheres and Earth System, and College of Marine Life Sciences, Ocean University of China, Qingdao, China; 2 Laboratory for Marine Ecology and Environmental Science, Qingdao Marine Science and Technology Center, Qingdao, China; 3 Key Laboratory of Evolution & Marine Biodiversity (Ministry of Education), and Institute of Evolution & Marine Biodiversity, Ocean University of China, Qingdao, China; 4 Marine Agriculture Research Center, Tobacco Research Institute of Chinese Academy of Agricultural Sciences, Qingdao, China; The Ohio State University, Columbus, Ohio, USA

**Keywords:** dimethylsulfoniopropionate (DMSP), dimethyl sulfide (DMS), deep sea, novel pathway, sulfur cycle

## Abstract

The microbial cycling of dimethylsulfoniopropionate (DMSP) and dimethyl sulfide (DMS) constitutes a crucial component of the global sulfur cycle, influencing climate regulation through the release of climate-active gases. Since DMSP production has historically been attributed to planktonic algae, research on its cycling has consequently centered on the surface ocean. However, the continuous discovery of bacterial DMSP synthesis genes (*dsyB*, *mmtN,* and *dsyGD*) has confirmed that the DMSP/DMS cycling process may also exist in the aphotic deep-sea environment. Further supporting this, the recently identified hydrogen sulfide (H_2_S)/methanethiol (MeSH)-dependent DMS-producing enzyme MddH, widespread among marine bacteria, may represent a significant source of DMS. This review integrates emerging insights into the ecological roles, environmental distribution, and microbial metabolism of these organic sulfur compounds, with a specific emphasis on deep-sea environments. We further discuss the distribution of key microbial taxa and functional genes involved in the biosynthesis and degradation of DMSP and DMS across deep-sea water columns and sediments. Collectively, this review highlights the existence of an active microbial DMSP/DMS cycling network in the deep ocean that remained overlooked until recently, with profound implications for global biogeochemical cycles and climate.

## INTRODUCTION

The organic sulfur cycle, centered around dimethylsulfoniopropionate (DMSP) and its climatically active metabolite dimethyl sulfide (DMS) (1), is a complex and dynamic component of global sulfur cycling, involving biochemical transformations of diverse organosulfur compounds. Since algae were considered to be the dominant producers of abundant DMSP in the ocean (2), research has primarily focused on the upper ocean layers, where massive DMSP production by algae and its subsequent microbial conversion to DMS were established as key processes. While the significance of DMSP/DMS-related organic sulfur cycling has been recognized, it was largely limited to the euphotic zones. The vast dark ocean, representing over 90% of the ocean volume (3), has traditionally been viewed as a major reservoir for exported organic matter, where organosulfur compounds are rapidly processed and largely remineralized back to sulfate through microbially mediated transformations (4, 5). However, intermediate transformations within the organic sulfur pool and release of DMS in the deep oceans remain poorly understood (5). Recent discoveries from the deep sea, including hadal trenches and abyssal sediments, have revealed not only detectable concentrations of DMSP in these extreme environments, but more importantly, an unexpected genetic and metabolic capacity for organic sulfur cycling among deep-sea microbes (6–9). This emerging evidence, including the direct detection of DMSP and the widespread occurrence of genes and metabolic pathways involved in the conversion of organic sulfur compounds, suggests that the deep ocean represents an active, metabolically distinct environment where microbes drive organosulfur transformation. This review aims to summarize these recent discoveries, delineating how deep-sea microbes drive a unique sulfur cycle network and elucidating the potential influence of this previously hidden process on global sulfur fluxes.

## ECOLOGICAL IMPORTANCE OF DMSP/DMS CYCLING

**Fig 1:**
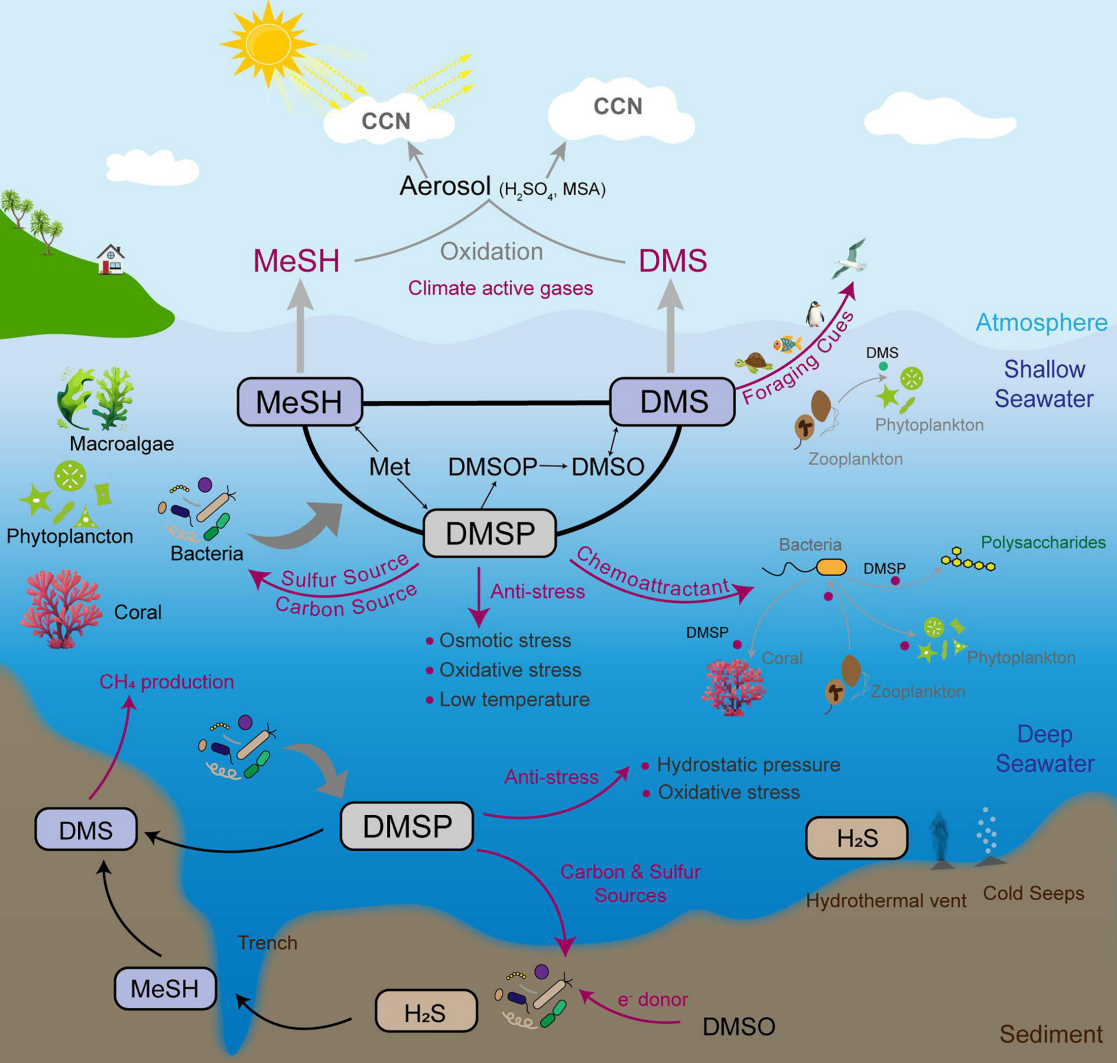
Ecological and physiological roles of DMSP, DMS, and related organic sulfur compounds. The schematic illustrates the major transformation pathways and potential roles of DMSP and its volatile derivatives, DMS and MeSH. DMSP is synthesized by marine bacteria, phytoplankton, macroalgae, and corals. Degradation of DMSP produces DMS and MeSH, which can be emitted into the atmosphere. Subsequent atmospheric oxidation of these gases generates sulfate and methanesulfonic acid (MSA) aerosols, which function as cloud condensation nuclei (CCN), thereby influencing Earth’s climate. Beyond its role in the sulfur cycle, DMSP and its metabolites serve critical ecological functions, including providing sulfur and carbon for marine bacteria, acting as protective compounds against various environmental stresses (e.g., osmotic and oxidative stress and hydrostatic pressure), and facilitating trophic interactions as infochemicals (e.g., chemoattractants). The diagram also denotes linkages to other processes such as methane (CH_4_) production and carbon polymer turnover.

The DMSP/DMS cycling involves active interconversion among DMSP, DMS, methanethiol (MeSH), dimethyl sulfoxide (DMSO), and other sulfur compounds (10). This cycling process sustains critical processes that span global climate regulation, biogeochemical cycling of carbon and sulfur, and a multitude of ecological interactions from the cellular to the ecosystem scale (Fig. 1).

### Climate regulation

The climatic relevance of the marine organic sulfur cycle has long been a primary driver of interdisciplinary research interest, with focus on the climatically active DMS as the principal source of reduced sulfur to the marine atmosphere (1). In the 1980s, the CLAW hypothesis (11) first proposed a climate feedback loop wherein biologically produced DMS from the ocean is oxidized in the atmosphere to form methanesulfonic acid (MSA) and sulfate aerosols. These aerosols contribute to climate regulation by directly backscattering solar radiation and, more significantly, by acting as cloud condensation nuclei (CCN), thereby enhancing cloud albedo and exerting a net cooling effect on the Earth’s surface (12, 13). Current estimates suggest the magnitude of this DMS-mediated cooling could be comparable to the warming effect of anthropogenic carbon dioxide (CO₂) emissions (14), underscoring the critical importance of elucidating marine DMS sources and sinks in the context of global climate change.

Beyond the long-recognized climatic effects of DMS, recent studies have illuminated the previously underestimated role of another volatile organic sulfur compound, MeSH, in atmospheric chemistry. MeSH emissions are now estimated to account for approximately 16% of marine volatile organic sulfur emissions, which are primarily composed of DMS and MeSH (15). Due to its rapid and efficient oxidation to sulfur dioxide (SO_2_), a key precursor for sulfuric acid (H_2_SO_4_) aerosol formation, MeSH substantially contributes to atmospheric sulfur loading (15). Investigations in the Southern Ocean revealed that MeSH emissions can increase atmospheric sulfur aerosols by 30%–70%, suggesting a potent role of MeSH in reinforcing climate cooling effects (16). Nevertheless, MeSH production and flux remain far less understood than those for DMS, representing a critical gap in our climate models.

### Biogeochemical cycling

As the precursor of DMS and MeSH (17), DMSP accounts for roughly half of the sulfur assimilated by marine primary producers (4, 18), supporting 3%–15% of the microbial carbon demand and 30%–100% of the microbial sulfur demand (2, 19). For ubiquitous bacterial clades deficient in sulfate assimilation pathways, such as SAR11 and SAR86, DMSP acts as an essential source of reduced sulfur to sustain their growth (20, 21). The catabolic fate of DMSP determines the flow of its sulfur through the ecosystem. Approximately 10% of DMSP is converted to DMS through the cleavage pathway, while 75% is routed to MeSH and intermediates like methylmercaptopropionate (MMPA), which are ultimately incorporated into the microbial food web (14, 22–24). The high flux and remarkably fast turnover times of DMSP confer upon it an indispensable role in marine nutrient cycles (25). Furthermore, DMSP and DMS participate in the carbon cycle through indirect yet consequential mechanisms. Recent work has revealed that DMSP enhances bacterial chemotaxis toward polysaccharides, potentially accelerating the degradation of carbon polymers and thus influencing the oceanic carbon pump (26). Equally noteworthy is the role of DMS as a precursor to greenhouse gas methane (CH₄). The DMS-to-CH_4_ conversion commonly occurred across various marine ecosystems, particularly in anoxic sediments, mediated by some methanogenic and methylotrophic microbes (27–30), representing a critical and overlooked link between the global sulfur and methane cycles.

### Stress protection and chemo-signaling

For different organisms, DMSP and its metabolites play key physiological roles and act as infochemicals. DMSP has long been recognized as a compatible solute, functioning as an osmoprotectant in marine algae and bacteria (31, 32). Notably, its significance may be particularly pronounced in deep-sea biomes, where studies in the Mariana Trench suggest that DMSP helps bacteria cope with extreme hydrostatic pressure (6). Additionally, the oxidation of DMSP into a novel member of the organic sulfur cycling, dimethylsulfoxonium propionate (DMSOP), has been proposed as a mechanism contributing to oxidative stress mitigation (33). The ecological influence of these compounds is further amplified by their role as chemical signals. DMSP and its degradation products, including DMS and acrylate, mediate complex interactions within marine communities. They act as powerful chemoattractants for marine bacteria and herbivorous protists, influencing algal-bacterial associations and microbial colonization processes (34, 35). Furthermore, DMSP or DMS serve as foraging cues for zooplankton and higher trophic levels (e.g., turtle, fish, penguin, and seagull) (36–40), thereby shaping trophic dynamics and energy transfer in the pelagic ecosystem.

## DISTRIBUTION OF DMSP/DMS-RELATED ORGANIC SULFUR COMPOUNDS IN THE DEEP SEA

The direct detection of DMSP and related compounds in the deep ocean suggests that there could be active microbial metabolic processes of these organic sulfur compounds. While their concentrations are generally lower than in productive surface waters, their persistence and dynamic distribution patterns point to active *in situ* production and consumption, revealing a previously hidden layer of microbial activity.

### DMSP in the deep water column and sediments

**Fig 2:**
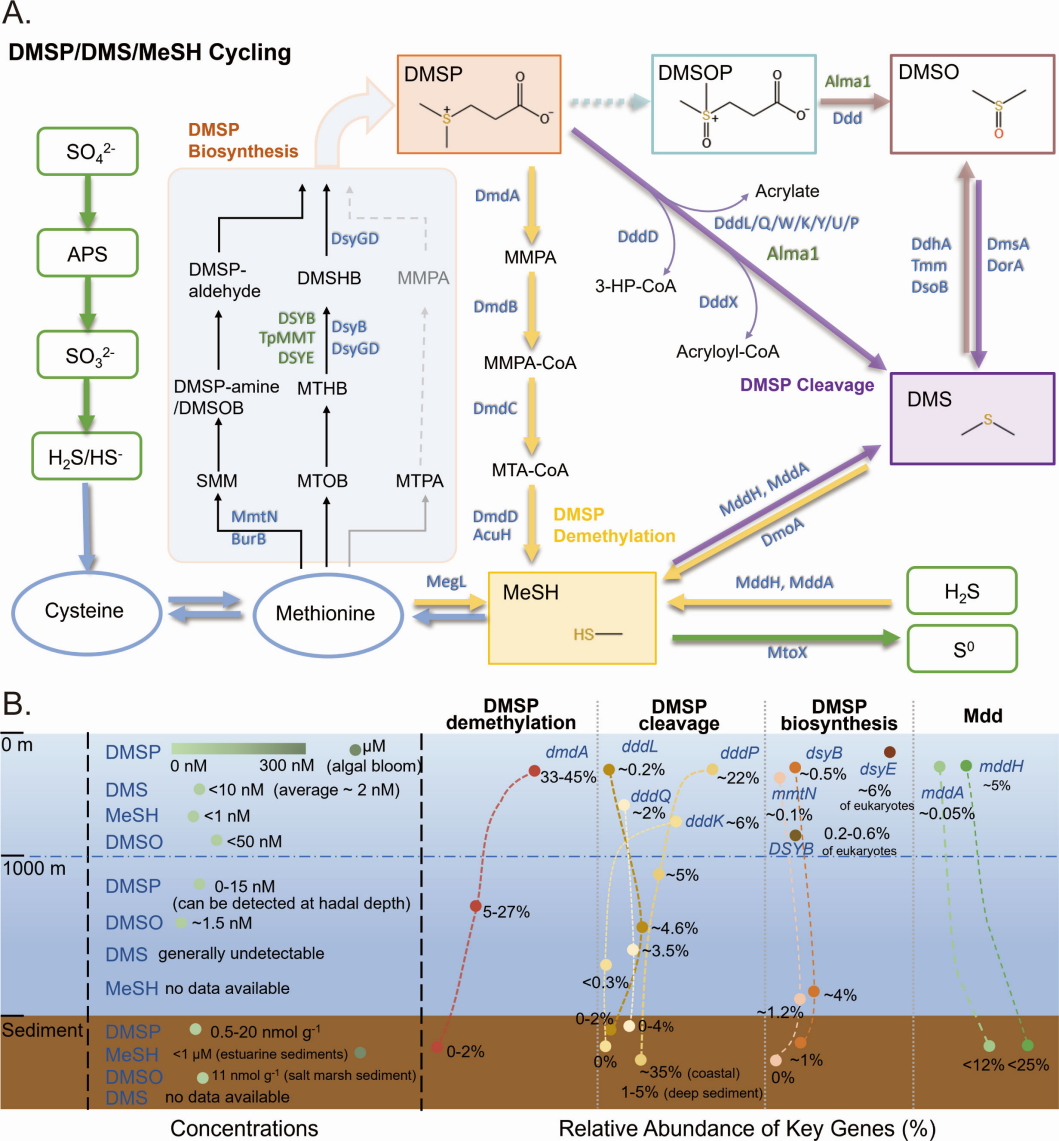
DMSP/DMS/MeSH cycling pathways and abundance of key genes. (**A**) An integrated view of the DMSP/DMS/MeSH cycling pathways and genes. The diagram summarizes the major biosynthesis and degradation pathways of DMSP/DMS and its interconnection with the broader sulfur cycle. The core depicts DMSP biosynthesis from methionine via multiple pathways (transamination, methylation, and decarboxylation), highlighting key enzymes such as DsyB, DSYB, MmtN, and the recently identified bifunctional synthase DsyGD. DMSP degradation proceeds primarily via the demethylation pathway (key enzyme DmdA) to MeSH or the cleavage pathway (via diverse Ddd lyases and Alma1) to DMS. Alternative routes include the MddH/MddA-mediated production of DMS from sulfide/methanethiol (H_2_S/MeSH), the reduction of DMSO to DMS by DMSO reductases (DmsA and DorA), and the oxidation of DMS to DMSO by DdhA/Tmm/DsoB. The schematic also shows the oxidation of DMSP to DMSOP and the conversion of MeSH to sulfane sulfur (S^0^) by methanethiol oxidase (MtoX). The abundant sulfate (SO_4_^2−^) in the ocean can be reduced to H_2_S/HS^−^, which is subsequently incorporated into sulfur-containing amino acids (methionine and cysteine). Among them, methionine serves as a precursor for both DMSP and MeSH. The Mdd process, which catalyzes the production of DMS from H₂S, represents a critical link connecting the organic and inorganic sulfur cycles. Blue font indicates enzymes of microbial origin, and green font indicates enzymes of algal origin. Dashed lines represent pathways for which key enzymes have not yet been reported. For the color of the box and arrows: Green: inorganic sulfur cycling. Blue: sulfur-containing amino acid transformation. Orange: DMSP and its production. Yellow: MeSH and its production. Purple: DMS and its formation. Brown: DMSO production. Light blue: DMSOP and its biosynthesis. APS: adenosine 5′-phosphosulfate, DMSOB: 4-dimethylsulfonio-2-oxobutyrate, SMM: *S*-methyl-methionine, MTOB: 4-methylthio-2-oxobutyrate, MTHB: methylthiohydroxybutyrate, DMSHB: 4-dimethylsulfonio-2-hydroxybutyrate, MTPA: 3-methylthiopropylamine, MMPA: methylmercaptopropionate, and MTA-CoA: methylthioacrylyl‐CoA. (**B**) Schematic diagram of vertical changes in key compound concentrations and relative abundances of key metabolic genes during the DMSP/DMS/MeSH cycle. The relative abundance of genes refers to normalization based on single-copy marker genes of eukaryotes or prokaryotes.

In the surface seawater, the concentrations of DMSP range from 0 to 300 nM in most oceanic regions (as reviewed by Zhang et al. [41]) and can reach 1–2 μM levels during algal blooms (Fig. 2) (41, 42). Previous studies suggest that DMSP concentrations decrease sharply with seawater depth, with the majority of production and turnover occurring in the upper water column (25), resulting in relatively low concentrations in deeper seawater (for instance, 0.5–22 nM at depths between 70 and 690 m in the Ross Sea, and less than 1 nM below 100 m in the South Pacific Ocean and eastern Indian Ocean) (43–45). Although measurements of DMSP content in the deep sea remain limited, growing evidence confirms that DMSP is not only present but can exhibit complex vertical and spatial patterns in the deep ocean. Crucially, DMSP can be detected throughout the water column, even at hadal depths. In the Mariana Trench, DMSP levels remained remarkably stable (0.96–2.39 nM) from 200 m down to 10,000 m, suggesting a balance between continuous supply and consumption far from surface sources (6). In the Yap Trench, DMSP concentrations ranged from 2.0 to 15.2 nM for the 0–6,000 m range and reached the highest at 4,000 m depth (46).

While in the deep-sea sediments, the DMSP content is typically 2–3 orders of magnitude higher than that in seawater per equivalent mass (mL vs. g) (6, 7, 9, 47). In the Challenger Deep sediments, DMSP concentrations ranged from 3.26 to 9.66 nmol g^−1^, while in the South China Sea (SCS), subseafloor sediments contained 0.56–2.08 nmol g^−1^, with much higher levels (11.25–20.90 nmol g^−1^) in the surface sediments (7, 9). The vertical variation of DMSP concentration with sediment depth is closely related to the deposition and microbial process: it decreased sharply with depth under continuous sedimentation regimes (e.g., SCS), whereas heterogeneous sedimentation processes in hadal trenches can result in complex, fluctuated depth distributions (7, 9). The abundance of DMSP in these deep-sea sediments implies the existence of active and likely diverse microbial metabolic processes.

### DMS, DMSO, and MeSH in the dark ocean

The distribution of DMS in the deep sea is characterized by rapid biological consumption and generally low concentrations, which are up to an order of magnitude lower than that of DMSP (41, 43). While the global average surface seawater DMS concentration is around 2.26 nM (48), it typically becomes undetectable below 200 m (43, 45, 49, 50). However, notable exceptions occur, particularly in high-latitude regions like the Ross Sea, Southern Ocean, and Weddell Sea, where the export of phytoplankton biomass and subsequent bacterial activity in deeper waters can lead to elevated DMS concentrations, decoupling its production from immediate surface production (43, 51, 52). The biological consumption of DMS contributes to the pool of DMSO, which is typically present at low nanomolar concentrations in deep seawater (43, 53). This highlights the active cycling between the reduced and oxidized forms of dimethyl sulfur even in the aphotic zone.

Among these volatile compounds, MeSH is more rapidly consumed and exhibits faster turnover times, leading to dissolved concentrations often below 1 nM in surface waters and making its quantification in the deep sea and sediments exceptionally challenging (15, 16, 23). Notably, MeSH has been widely detected in mid-ocean ridge hydrothermal fluids, reaching millimolar levels in some low-temperature (<200°C) mixed fluids (54). These hydrothermal fluids inject significant quantities of MeSH into the deep ocean, representing a potentially major, yet poorly quantified, source of organic sulfur that influences microbial metabolic strategies in near-seafloor and plume habitats.

## DMSP/DMS CYCLING PATHWAYS AND NOVEL MECHANISMS

The microbial cycling of DMSP and DMS involves a complex network of interconversion pathways between different sulfur compounds (Fig. 2; Table S1). Previously, the DMSP lyases and demethylases that received the most attention were predominantly identified in bacteria from surface ocean environments where phytoplankton produce substantial amounts of DMSP. However, the identification of bacterial DMSP biosynthesis genes (*dsyB* and *mmtN*) has demonstrated that DMSP production is not only restricted to photosynthetic organisms in surface waters but can also occur in heterotrophic bacteria, providing an *in situ* source of DMSP in the deep ocean. Furthermore, a series of the MeSH-dependent DMS production (Mdd) enzymes recently identified from deep-sea bacteria indicate that H_2_S and MeSH in deep-sea environments and sediments may also serve as important sources of DMS. These novel discoveries have reshaped our understanding of DMSP/DMS cycling processes, revealing previously unrecognized pathways and a remarkable diversity of enzymes.

### DMSP biosynthesis: from phytoplankton-dominated surface production to bacterial synthesis in the deep sea

The synthesis of DMSP was previously considered an exclusive capability of marine photosynthetic organisms, primarily phytoplankton and macroalgae. A fundamental shift in this understanding came with the discovery of bacterial DMSP synthesis (55). The identification of the key DMSP synthase, DsyB, in the marine bacterium *Labrenzia aggregata* LZB033, represented the first synthase identified in all DMSP-synthesizing organisms (55). This finding not only promoted research into the genetic machinery for DMSP production but also provided crucial evidence supporting the possibility of active DMSP synthesis in aphotic deep sea, where algal production is absent.

Currently, three DMSP biosynthesis pathways (the transamination, methylation, and decarboxylation pathways) have been identified, all utilizing methionine (Met) as the precursor. DsyB catalyzes the conversion of 4-methylthio-2-hydroxybutyrate (MTHB) to 4-dimethylsulfonio-2-hydroxybutyrate (DMSHB) in the transamination pathway (55). Homologs of *dsyB* were subsequently found in eukaryotic algae and corals, encoding a functional DMSP synthase, DSYB (56). Additionally, TpMMT (*Thalassiosira pseudonana* MTHB-methyltransferase) in diatoms was also capable of catalyzing this key step in the transamination pathway (57). Most recently, a novel and structurally distinct bifunctional DMSP synthase, DsyGD, was identified in the rhizobacterium *Gynuella sunshinyii* and some filamentous cyanobacteria (58). The uniqueness of DsyGD lies in its fusion of two catalytic domains: the N-terminal DsyG domain methylates MTHB to DMSHB, and the C-terminal DsyD domain subsequently decarboxylates DMSHB to yield DMSP (58). Furthermore, homologs of the DsyG domain alone, named DSYE, have been identified in high-DMSP-producing algal groups such as Pelagophyceae (58). The discovery of DsyGD and DSYE has expanded our understanding of the genetic diversity of DMSP synthesis across different DMSP-producing organisms.

In the methylation pathway, the first key gene reported was *mmtN*, which encodes an enzyme that converts methionine to S-methyl-methionine (SMM) (47). BurB, another enzyme that catalyzes this step, has been reported exclusively in *Burkholderia thailandensis* (59). In contrast to the well-characterized transamination and methylation pathways, the key enzyme in the decarboxylation pathway remains unknown; thus, whether this pathway exists in marine bacteria is yet to be elucidated.

### DMSP catabolism: the growing diversity of DMSP lyases

The two major pathways of DMSP degradation, demethylation and cleavage pathway, are catalyzed by the enzyme encoded by *dmdA* and a series of *ddd* genes (along with Alma1 in algae), respectively. In the demethylation pathway first reported in *Ruegeria pomeroyi* DSS-3, DmdA catalyzes the conversion of DMSP to MMPA, which is subsequently transformed into MeSH through the action of DmdBCD and AcuH (60–62). The *dmdA* gene family is categorized into clades A, B, C, D, and E and is further divided into 14 subclades (63, 64). In the cleavage pathway, nine distinct *ddd* genes have been identified (*dddL/Q/W/K/Y/U/P/D/X*). Six of these Ddd enzymes (*dddL/Q/W/K/Y/U*) belong to the cupin superfamily, while DddP, DddD, and DddX are from the M24 peptidase family, type III acyl CoA transferase family, and acyl-CoA synthetase superfamily, respectively (65). DddL/Q/W/K/Y/U/P cleave DMSP to form acrylate and DMS (65), while DddD and the recently discovered DddX are unique in converting DMSP to 3-hydroxypropionyl-CoA (3-HP-CoA) and acryloyl-CoA, respectively, in addition to DMS (66, 67). In algae, Alma1 is the only known DMSP lyase and shares no sequence similarity with bacterial *ddd* genes (68). Intriguingly, many of these DMSP lyases (Ddd proteins and Alma1) can also cleave DMSOP to DMSO, employing similar catalytic mechanisms (69). The gene(s) responsible for oxidizing DMSP to DMSOP, however, remains unknown.

### Mdd pathway: alternative DMS source identified in deep-sea bacteria

For decades, DMSP cleavage via diverse Ddd enzymes was considered the principal source of oceanic DMS. However, our recent discovery challenged this view by focusing on a previously overlooked process, the conversion of hydrogen sulfide (H_2_S)/MeSH to DMS (70). A novel DMS-producing enzyme widespread in the ocean, MddH, which catalyzes the formation of DMS from H_2_S and MeSH, was identified in *Halomonas* sp. EF61 isolated from Mariana Trench seawater (~3,600 m) (70). Although Mdd pathway was first identified in 2015, the initially reported key enzyme MddA was found to be predominantly present in terrestrial environments with low abundance in the ocean and thus was not considered a potentially significant marine source of DMS (71–73). Unlike *mddA,* which mainly existed in terrestrial soil or freshwater bacteria (71, 72), *mddH* showed broader distribution in marine bacteria and higher catalytic efficiency than MddA in generating DMS from both H_2_S and MeSH (70). The discovery of MddH underscores that H_2_S/MeSH-dependent DMS production represents not only a potential overlooked source of DMS but also a direct metabolic connection between the inorganic sulfur pool (H_2_S) and the organic sulfur cycle (DMS). Subsequently, identification of two novel Mdd enzymes from an actinomycete in the Mariana Trench, MddM1 and MddM2 (74), expands the diversity of Mdd enzymes and also highlights that diverse microbial taxa in the deep sea may harbor yet unidentified Mdd enzymes.

Importantly, the deep-sea environment may create favorable conditions for Mdd-mediated DMS production. In anaerobic environments, such as oxygen-deprived deep sediments, sulfate-reducing bacteria convert the abundant sulfate into H_2_S. Extreme deep-sea settings like hydrothermal vents also harbor high concentrations of H_2_S (75). Furthermore, MeSH can be sourced not only from DMSP demethylation but also directly from methionine via methionine γ-lyase (MegL) in many bacteria (76, 77). These sources may thereby provide substantial precursors for the production of MeSH and DMS in the deep ocean.

### Other metabolic pathways for DMS and MeSH

In addition to DMSP-dependent and Mdd pathways, DMS can also be produced via the reduction of DMSO catalyzed by DMSO reductases (DMSORs). Two types of DMSOR have been described in *Escherichia coli* and *Rhodobacter* species, referred to as the Dms and Dor types, respectively (78, 79). In environments with high DMSO concentrations, like some salt marsh sediments, this reduction can be a relevant DMS source and may couple with DMS-dependent methanogenesis (80). For the consumption of DMS, DMS monooxygenase (DmoA) oxidizes DMS to MeSH (81); DMS dehydrogenase (DdhA), assimilatory DMS *S*-monooxygenase (DsoB), and trimethylamine monooxygenase (Tmm) are involved in the oxidation of DMS to DMSO (82–84). Furthermore, MeSH can be converted to formaldehyde and H_2_S (or sulfane sulfur) through MeSH oxidase MtoX in many bacteria (85, 86).

## DISTRIBUTION OF DMSP/DMS CYCLING GENES AND TAXA IN DEEP-SEA ENVIRONMENTS

As genes involved in DMSP/DMS cycling continue to be identified and pathways are better resolved, they have become key markers indicating the metabolic potential of DMSP, DMS, and other related organic sulfur compounds. Linking these functional genes to their distribution and taxonomic origins across marine environments enables the identification of dominant microbial processes. In particular, variations in gene abundance and host composition along environmental gradients reveal potential hotspots of DMSP/DMS cycling and delineate clear vertical patterns from surface waters to the deep ocean and sediments (Fig. 2B).

### DMSP production

As the most abundant bacterial DMSP synthesis gene in marine environments, *dsyB* is widely distributed among marine Alphaproteobacteria, particularly within the Rhodobacterales order (47). Up to 0.5% of seawater bacteria were predicted to harbor *dsyB* according to metagenomic analyses of the Tara Oceans and Global Ocean Sampling projects (47). Quantitative PCR (qPCR) assays targeting *dsyB* in seawater samples revealed absolute abundances ranging from 10^0^ to 10^3^ copies per milliliter (87–89). By contrast, in diverse marine sediments, the relative abundance of *dsyB* can reach approximately 1% of the bacterial community, with estimated absolute abundances as high as 10^8^ copies per gram sediment (47). These findings indicate that bacteria serve as important producers of DMSP in marine sediment ecosystems. The *mmtN* gene also exists in several alphaproteobacterial genera (e.g., *Thalassospira*, *Novosphingobium*, and *Ponticaulis*) and in a few Actinobacteria and Gammaproteobacteria; however, its absolute abundance is normally one to three orders of magnitude lower than *dsyB* in both seawater and sediment (47, 87, 89). Other bacterial DMSP synthesis genes, such as *burB* and the recently described *dsyGD* (58, 59), have only been found in a narrow range of bacterial taxa and are thus likely to make a relatively minor contribution to environmental DMSP pools compared to *dsyB* and *mmtN*.

The hadal zone of the Mariana Trench represents a key environment for investigating DMSP synthesis in the deep sea. Below 2,000 m, the abundance of the *dsyB* gene increases significantly with depth (6). Its copy number and relative abundance reach a maximum at 10,500 m (3.95 × 10^6^ copies L^−1^; 4.03% of bacterial community estimated based on single-copy marker normalization), substantially exceeding values in the trench’s surface waters (2.61 × 10^5^ copies L^−1^; 0.78%–0.98%) (6). Although less prevalent than *dsyB*, *mmtN* was identified in as much as ~1.22% of bacteria in seawater at 8,000 m depth (6). Metagenomic analyses indicate that the total relative abundance of bacterial DMSP synthesis genes (*dsyB* and *mmtN*) can reach 2.58%–5.25% in waters below 4,000 m, compared to only ~1% in surface waters (6). This vertical enrichment is coupled with a shift in the microbial community, with *dsyB*-harboring taxa like *Pseudooceanicola* and *Roseovarius* being significantly enriched in deeper layers (≥ 4,000 m) (6).

In deep-sea sediments, *dsyB* is often the sole detectable DMSP synthesis gene via qPCR and metagenomes, with abundances ranging from 10^2^ to 10^3^ copies g^−1^ (7, 9). Its vertical profile frequently mirrors that of DMSP concentration, decreasing sharply with depth in continuously sedimentation regimes like the SCS, but exhibiting a more complex, fluctuated pattern in hadal trenches influenced by heterogeneous sedimentation processes (7, 9). The discovery of diverse DMSP-producing bacterial isolates (e.g., *Marinobacter*, *Alcanivorax*, *Qipengyuania*, *Dietzia*, *Brachybacterium*, and *Mammaliicoccus*) that lack known synthesis genes further suggests a reservoir of uncharacterized genetic diversity for DMSP biosynthesis in deep-sea sediments (7, 9).

In contrast, algal DMSP synthesis genes are predominantly confined to surface waters. Genes encoding the dominant enzyme DSYB, primarily found in haptophytes and dinophytes, were more widely detected than the newly characterized *DSYE* genes from pelagophytes and chlorophytes (56, 58). Furthermore, *DSYB* transcript abundances were approximately twofold higher than those of *DSYE* (58). By comparison, transcripts of the diatom-specific enzyme TpMMT were consistently one to two orders of magnitude lower than those of both algal DSYB and DSYE (58). Nevertheless, *DSYB*, *DSYE,* and *TpMMT* genes in algae and *dsyB*, *mmtN*, and *dsyGD* (*dsyG*) in diverse bacteria are currently reliable reporters to predict DMSP-producing potential in both organisms and environments.

### DMSP degradation

Unlike bacterial DMSP biosynthesis, which predominates in aphotic seawater and sediments rather than algal DMSP production, bacteria mediate the primary degradation of DMSP across diverse marine environments. DmdA was widely present in the Roseobacteraceae and SAR11, which were among the most abundant bacterial groups in surface seawaters, as well as in SAR116 and Gammaproteobacteria (90, 91). Metagenomic analysis of the GOS data set estimates that approximately 58% (±9%) of marine bacteria possess *dmdA* (90). Quantitative PCR (qPCR) analyses indicate that the absolute abundance of *dmdA* can reach 10^7^ copies L^−1^ in surface seawater (88, 89), with the subclades C/2 and D/1 being the most prevalent in marine systems (63, 64). Among *ddd* genes, *dddP* is generally considered the most abundant in surface seawater, followed by *dddQ*, *dddL*, and *dddD* (89). These *ddd* genes are distributed across diverse Alphaproteobacteria (Roseobacteraceae, SAR11, SAR116, and Rhizobiales) and Gammaproteobacteria (Pseudomonadales and Oceanospirillales) (41). The *dmdA* gene is typically several-fold more abundant than *dddP*, which aligns with the established view that DMSP demethylation is the dominant degradation pathway (89).

In the deep water column of the Mariana Trench, the catabolic potential showed similar patterns as observed in surface oceans, with higher abundance of *dmdA* and *dddP* in general and dominant DMSP degradation groups such as SAR11, Roseobacteraceae, and SAR116 (6). Vertically, a substantial proportion (14.28%–36.88%) of bacteria predicted to carry DMSP catabolic genes even at depths of 2,000–8,000 m of the Mariana Trench (6). At these depths, both *dmdA* and *dddP* maintained high abundances, while certain lyase genes (*dddQ*, *dddL*, and *dddD*) can exhibit even greater abundances in specific deep-water layers than at the surface (6).

However, studies on deep-sea sediments have revealed a distinct distribution of bacterial DMSP catabolic genes relative to seawater. In the Challenger Deep sediments, *dddP* turned out to be the most abundant DMSP catabolic gene, reaching ~10^4^ copies g^−1^, while *dmdA* was undetectable (7). This aligns with findings from Eastern China Marginal Seas sediments, where *dddP* consistently outnumbered *dmdA* (92). Conversely, other studies report the presence of only *dmdA* in Mariana Trench surface sediments (10^1^–10^3^ copies g^−1^) or a sharp decrease of *dmdA* with depth in SCS sediments, with *dddP* below detection (6, 9). These divergent findings point to a clear heterogeneity in the distribution of DMSP-degrading genes among deep-sea sediments, the underlying causes of which remain to be fully elucidated. Furthermore, the discovery of *ddd* genes in anaerobic Anaerolineales metagenome-assembled genomes (MAGs) and the isolation of diverse DMSP-catabolizing Actinomycetia from Challenger Deep sediments with unknown degradation genes highlight the vast unexplored diversity of deep-sea sediment microbes involved in DMSP turnover (7).

### Mdd pathway

MddH homologs were present in diverse bacterial taxa, primarily within the Gammaproteobacteria and Alphaproteobacteria, but also extending to some Betaproteobacteria, Deltaproteobacteria, Acidobacteria, and Bacteroidetes (70). In contrast, previously characterized MddA was mostly found in Actinobacteria, Rhizobiales from Alphaproteobacteria, and was far less common in Gammaproteobacteria compared to MddH (70). The percentage of *mddH* ranged 0.09%–5.2% (with an average of 2.19% ± 0.93%), while *mddA* was significantly less abundant than *mddH* in Tara Ocean metagenomes (0.04% ± 0.07%) (70). The relative abundance of *mddH* can reach ~15% in coastal sediments, while that for *mddA* was only 0.39%–3.34% (70). Phylogenetically, *mddH* genes in sediments are primarily associated with Gammaproteobacteria, although they are also detected in Acidobacteria and Deltaproteobacteria. Conversely, *mddA* in sediments originates from a broader range of taxa, including Ignavibacteriae, Nitrospirae, Planctomycetes, and Bacteroidetes (70). Considering that these sediments may contain higher levels of MeSH and H_2_S (even at mM levels), which can be even more abundant than DMSP that support DMSP lyase activity, marine sediments can be potential hotspots for MddH-driven DMS production.

Further study on different marine sediments revealed that Mdd activity can be detected in either near-shore, pelagic, deep-sea, and cold seep sediments. The abundance of *mdd* genes can reach 24.55%–26.73% in pelagic deep-sea sediments from the Kuroshio-Oyashio Extension region (at water depth of ~5,980 m) (93). Several Bacteroidota and Bacillota isolates may possess unknown Mdd enzymes (93). A newly developed high-throughput degenerate qPCR chip for profiling DMSP/DMS cycling (DSMG-Chip) revealed abundant *mddH* in the Pacific Ocean at water depths of 1,000 m and 2,000 m (8). In sediments from the Haima cold seep, Yokosuka hydrothermal vent, and the Mariana Trench, abundant *mdd* genes (primarily *mddA*) were detected, with their abundance generally decreasing as sediment depth increased (8). These findings indicate that active Mdd processes may be prevalent in extreme deep-sea environments such as cold seeps and hydrothermal vents, whereas the potential existence of novel *mdd* across different deep-sea environments needs further in-depth investigations.

### Other DMSP/DMS metabolic pathways

Beyond the principal DMSP and DMS production pathways discussed above, research on the distribution of other genes within this cycling network remains relatively limited. Despite this, several of these genes involved in this organic sulfur cycling process demonstrate remarkably high abundance in marine environments. For instance, *megL*, which encodes a methionine γ-lyase that directly produces MeSH from methionine, has been widely identified in MAGs from diverse habitats such as the brackish waters of the Caspian Sea and deep groundwater. This gene is found in various Proteobacteria and Firmicutes, as well as in archaea belonging to the phylum Halobacteriota (77). Genes encoding DmdBCD that convert MMPA to MeSH were also extremely abundant as found in marine sediments, with the relative abundance of *dmdB* in bacteria can reach 50% or even higher (7, 92).

While the mechanism and function of DMSOR (DorA and DmsA) have been extensively studied (94), their abundance in the marine environment and their contribution to the sulfur cycle have been less discussed. In Mariana Trench sediment metagenomes, *dorA* was identified in MAGs of Dehalococcoidia, Acidimicrobiia, and Gammaproteobacteria, predicted to exist in ~5% of sediment bacteria (7). For oxidation of DMS, *ddhA* from Actinobacteriota, Bacteroidota, and Chloroflexota exhibited a high relative abundance (9%–29%) in trench sediments, likely representing the predominant biological consumption pathway for DMS (7). The dominance of *ddhA* has also been observed in salt marsh and hydrothermal vent sediments (92, 95). In contrast, bacteria possessing *mtoX* accounted for 1%–12% in salt marsh, marginal seas, hydrothermal vent, and Mariana Trench sediment and were primarily detected within the Gammaproteobacteria (7, 92). In summary, current research on the complete suite of bacterial DMSP/DMS cycling genes has primarily been focused on sedimentary environments. There remains a notable lack of studies that systematically investigate the distribution and expression of these related genes, along with the identity of metabolically active taxa, across different depth profiles in the water column.

## FUTURE PERSPECTIVES IN DEEP-SEA DMSP/DMS CYCLING

Despite significant advances in understanding the microbial DMSP/DMS cycle in deep-sea environments, several critical challenges and knowledge gaps remain, pointing to promising avenues for future research. First, the discovery of novel enzymes such as MddH and DsyGD highlights that the genetic potential for organic sulfur cycling in the deep sea is far from fully characterized. The key enzymes for the DMSP decarboxylation biosynthesis pathway and the oxidation of DMSP to DMSOP remain unidentified. Future efforts should combine meta-omics with cultivation strategies to uncover this hidden genetic diversity and complete our understanding of the metabolic network. Additionally, the distribution of DMSP/DMS cycling genes and taxa exhibits clear heterogeneity across different deep-sea biomes (e.g., hadal trenches, cold seeps, hydrothermal vents, and abyssal sediments). The underlying environmental drivers, such as pressure, temperature, sulfur compound availability, and organic carbon flux, governing this heterogeneity are poorly understood. Systematic comparative studies across these deep-sea ecosystems covering diverse DMSP/DMS cycling genes are essential to identify the key factors shaping microbial DMSP/DMS cycling potential. Second, while gene abundance data provided insights into metabolic potential, they cannot directly quantify process rates or the actual contribution of specific pathways to sulfur fluxes. A significant challenge is to determine the *in situ* activity and the relative importance of the newly discovered Mdd pathway versus traditional DMSP cleavage in DMS production within dark, aphotic zones. Future studies should integrate rate measurements (e.g., using isotope tracers) with molecular analyses to bridge the gap between genetic potential and biogeochemical function. Finally, translating deep-sea microbial processes to global climate models remains a major challenge. The production and consumption of climate-active gases like DMS and MeSH in the deep ocean, and their eventual influence on the atmosphere, are poorly constrained. Future work should aim to quantify the deep ocean’s contribution to the global sulfur budget and evaluate its potential feedback on climate, particularly in the context of a changing ocean (96, 97).

## SUPPLEMENTAL MATERIAL

Table S1 mbio.01207-26-s0001.docx Summary of the origins, distribution, and related characteristics of key enzymes in the DMSP/DMS/MeSH cycle.

ASM does not own the copyrights to Supplemental Material that may be linked to, or accessed through, an article. The authors have granted ASM a non-exclusive, world-wide license to publish the Supplemental Material files. Please contact the corresponding author directly for reuse.
