# Supplementary material for: Novel insights into microbial DMSP/DMS cycling: from surface to deep ocean
Source: mBio. 2026 Jun 9;17(7):e01207-26. doi: 10.1128/mbio.01207-26 (PMC13360808; doi:10.1128/mbio.01207-26)
Supplement: Table S1 — Summary of the origins, distribution, and related characteristics of key enzymes in the DMSP/DMS/MeSH cycle. [file mbio.01207-26-s0001.docx]

**Table S1**. **Summary of the origins, distribution, and related characteristics of key enzymes in the DMSP/DMS/MeSH cycle.**

| **Protein** | **Pathways** | **Species Origin** | **Environmental Source** | **Relative abundance** | **Dominant Groups** | **Reference** |
| --- | --- | --- | --- | --- | --- | --- |
| **DmdA** | DMSP demethylation | *Ruegeria pomeroyi* DSS-3 | Coastal sea water | Surface seawater: ~33–45% of bacteria;  Mariana Trench seawater (2000–8000 m): ~5–27%; Deep sediment: ~1–2% or not detected. | Alphaproteobacteria (Rhodobacteraceae, SAR11) | (1) |
| **DmdB** | DMSP demethylation | *Ruegeria pomeroyi* DSS-3 | Coastal sea water | Surface seawater: Up to 61% of bacteria;  Sediment: variable at high level | Widespread across multiple bacterial phyla | (2) |
| **DmdC** | DMSP demethylation | *Ruegeria pomeroyi* DSS-3 | Coastal sea water | Surface seawater: 0–61% of bacteria;  Sediment: variable | Widespread across multiple bacterial phyla | (2) |
| **DmdD** | DMSP demethylation | *Ruegeria pomeroyi* DSS-3 | Coastal sea water | Eastern China marginal seawater and sediment: ~2% of bacteria;  Mariana Trench sediment: up to 25% | Not reported | (2) |
| **AcuH**  **(AcuK)** | DMSP demethylation | *Halomonas* HTNK1 | *Ulva lactuca* enrichment cultures | Eastern China marginal seawater: ~8% of bacteria; Mariana Trench sediment: up to 33% | Not reported | (3) |
| **DddL** | DMSP cleavage | *Sulfitobacter* EE-36 | Salt marsh | Global seawater: ~0.24% of bacteria;  Eastern China marginal seas: ~0.3%;  Mariana Trench seawater (4000–8000 m): up to 4.6%; Eastern China sediment: ~2%;  Mariana Trench sediment: not detected | Alphaproteobacteria (Rhodobacteraceae);  Gammaproteobacteria (Marinobacter) | (4) |
| **DddQ** | DMSP cleavage | *Ruegeria pomeroyi* DSS-3 | Coastal sea water | Global seawater: ~2.38% of bacteria;  Eastern China marginal seas: 6–14%;  Mariana Trench seawater (2000–8000 m): ~3.5%; Eastern China sediment: ~4%;  Mariana Trench sediment: not detected | Alphaproteobacteria (Rhodobacteraceae) | (5) |
| **DddW** | DMSP cleavage | *Ruegeria pomeroyi* DSS-3 | Coastal sea water | Global seawater: ~0.45% of bacteria;  Mariana Trench seawater: <0.35%;  Not detected in many seawater and sediment samples | Alphaproteobacteria (Rhodobacteraceae) | (6) |
| **DddK** | DMSP cleavage | *Pelagibacter* HTCC1062 | Coastal surface seawater | Global seawater: ~6.07% of bacteria;  Eastern China marginal seas: ~2–8%;  Mariana Trench seawater: <0.3%;  Eastern China sediment & Mariana Trench sediment: not detected | Alphaproteobacteria  (SAR11) | (7) |
| **DddY** | DMSP cleavage | *Alcaligenes faecalis* M3A | Salt marsh sediment | Global seawater: ~0.07% of bacteria;  Mariana Trench seawater: <0.13%;  Many environments: not detected | Betaproteobacteria; Gammaproteobacteria; Epsilonproteobacteria (*Alcaligenes*, *Arcobacter*, *Shewanella*) | (8) |
| **DddU** | DMSP cleavage | *Amylibacter cionae* H-12 | Sea squirt | Global seawater: ~1.44% of bacteria | Alphaproteobacteria (Rhodobacteraceae) | (9) |
| **DddP** | DMSP cleavage | *Roseovarius nubinhibens* ISM | Coastal sea water | Global seawater: ~22.3% of bacteria;  Eastern China marginal seas: 4.0–15.6%;  Eastern China sediment: up to 37.8%;  Mariana Trench sediment: 1.40–5.37% (10–700 cmbsf);  Mariana Trench seawater: 4.84% (average, 2000–8000 m), 6.48% (surface) | Alphaproteobacteria (Rhodobacteraceae, SAR116); Gammaproteobacteria; Betaproteobacteria; Actinobacteriota | (10) |
| **DddD** | DMSP cleavage | *Marinomonas* sp. MWYL1 | root surfaces of the salt marsh grass Spartina anglica | Eastern China marginal seas: up to 1.5% of bacteria;  Mariana Trench seawater (8000 m): ~1.61%;  Eastern China sediment: ~4%;  Mariana Trench sediment: not detected | Alphaproteobacteria (Rhodobacteraceae)  Gammaproteobacteria (*Halomonas*) | (11) |
| **DddX** | DMSP cleavage | *Psychrobacter* sp. D2 | Antarctic samples | Mariana Trench sediment: ~1% of bacteria | Alphaproteobacteria (Rhodobacteraceae); Gammaproteobacteria (*Psychrobacter*); Firmicutes (*Sporosarcina*) | (12) |
| **Alma1** | DMSP cleavage | *Emiliania huxleyi* | - | Surface and deep chlorophyll maximum: ~1.2% of eukaryotes | Haptophytes;  Dinoflagellates | (13) |
| **DsyB** | DMSP biosynthesis | *Labrenzia* *aggregata* LZB033 | Surface seawater East China Sea | Global ocean (Tara & GOS): ~0.5% of bacteria; Eastern China sediment & Mariana Trench sediment: ~1%;  Mariana Trench seawater (10500 m): ~4% | Alphaproteobacteria (Rhodobacterales, Rhizobiales, Rhodospirillales); Gammaproteobacteria | (14) |
| **MmtN** | DMSP biosynthesis | *Novosphingobium* sp. BW1 | Salt marsh surface sediment | Salt marsh sediment: ~0.1% of bacteria;  Mariana Trench seawater (8000 m): ~1.2%;  Many environments: not detected | Alphaproteobacteria (*Thalassospira*, *Roseovarius*, *Labrenzia*, *Novosphingobium*) | (15) |
| **DsyGD** | DMSP biosynthesis | *Gynuella sunshinyii* | salt marsh plants Carex scabrifolia and Spartina alterniflora | Global seawater (OM-RGC_V2): not detected | Cyanobacteria (Oscillatoriales); Gynuella | (16) |
| **BurB** | DMSP biosynthesis | *Burkholderia thailandensis* | Human pathogen | Marine environments: seldom detected | Burkholderia | (17) |
| **DSYB** | DMSP biosynthesis | the corals (*Acropora cervicornis*), diatoms (*F. cylindrus*), dinoflagellates (*Alexandrium tamarense*, *Lingulodinium polyedrum*, *Symbiodinium microadriaticum*) and prymnesiophytes (*Chrysochromulina tobin*, *Prymnesium parvum*) | - | Eastern China marginal seas: 0.2–0.6% of eukaryotes; Sediment: not detected | Haptophytes  Dinoflagellates  Diatoms  Corals | (18) |
| **TpMMT** | DMSP biosynthesis | *Thalassiosira pseudonana* CCMP1335 | - | Not reported | Diatom (*Thalassiosira*, *Phaeodactylum*) | (19) |
| **DSYE** | DMSP biosynthesis | *Ostreococcus prasinos* BCC99000  *Chroomonas mesostigmatica* CCMP1168 | - | SRF & DCM samples: ~6% of picoeukaryotes | Chloroarachniophyta; Chlorophytes; Pelagophytes | (16) |
| **MddH** | H_2_S/MeSH methylation | *Halomonas* sp. EF61 | Mariana Trench Seawater (3600 m) | Global seawater: ~5% of bacteria;  Sediment: up to 25% | Gammaproteobacteria | (20) |
| **MddA** | H_2_S/MeSH methylation | *Pseudomonas deceptionensis* M1^T^ | Antarctica marine sediment (depth unknown) | Global seawater: ~0.05% of bacteria;  Sediment: 0.4–12% | Alphaproteobacteria  Gammaproteobacteria  Bacteroidetes  Actinomycetota | (21) |
| **MddM1** | H_2_S/MeSH methylation | *Mycolicibacterium poriferae* ZYF656 | Mariana Trench seawater (9600 m) | Global seawater: 0.02–0.58% of bacteria;  Mariana Trench sediment: 1.78–3.8% | Actinomycetota  Proteobacteria  Chloroflexota | (21) |
| **MddM2** | H_2_S/MeSH methylation | *Mycolicibacterium poriferae* ZYF656 | Mariana Trench seawater (9600 m) | Global seawater & Mariana Trench sediment: not detected;  Marsh sediment: ~0.58% | Actinomycetota | (21) |
| **DdhA** | DMS oxidation to DMSO | *Rhodovulum sulfidophilum* SH1 | Surface mud from a tidal creek | Eastern China marginal seawater: 0.1–1.4% of bacteria;  Mariana Trench sediment: 9–29%;  Salt marsh: ~13.3% | Alphaproteobacteria, Gammaproteobacteria, Bacteroidia | (22) |
| **DsoB** | DMS oxidation to DMSO | *Acinetobacter* sp, 20B | Soil | Mariana Trench sediment: ~1.5% of bacteria | Gammaproteobacteria | (23) |
| **Tmm** | DMS oxidation to DMSO | *Ruegeria pomeroyi* DSS-3 | Coastal sea water | Eastern China marginal seawater: 0.1–1.4% of bacteria;  Mariana Trench sediment: 0.6–1.4%;  Salt marsh: ~2.1% | Alphaproteobacteria (Rhodobacteraceae, SAR11)  Gammaproteobacteria | (24) |
| **DmsA** | DMSO reduction to DMS | *Escherichia coli* | - | Not reported. |  | (25) |
| **DorA** | DMSO reduction to DMS | *Rhodobacter sphaeroides* 2.4.1^T^ | Freshwater environment | Mariana Trench sediment: ~5% of bacteria | Gammaproteobacteria MAGs Alphaproteobacteria | (26) |
| **DmoA** | DMS oxidation to MeSH | *Hyphomicrobium sulfonivorans* S1^T^ | Garden soil | Eastern China marginal seawater: ~1.0-3.4% of bacteria;  Mariana Trench sediment: ~10%;  Eastern China sediment: ~1%;  Salt marsh: ~0.5% | Alphaproteobacteria, Actinobacteriota, Gammaproteobacteria MAGs | (27) |
| **MtoX** | MeSH oxidation | *Hyphomicrobium* sp. VS | activated sewage sludge | Mariana Trench sediment: ~5-11.5% of bacteria; Seawater: 0.4–45.6%;  Freshwater: ~5.3%;  Soil: ~6.3% | Not reported | (28) |
| **MegL** | MeSH production from Met | *Pseudomonas ovalis* (=*Ps. putida*) IFO 3738 and other bacteria | Not mentioned | Not quantitatively reported; widespread in environments such as saltmarsh and Caspian Sea | Myxococcota; Gemmatimonadetes; Desulfobacterota; Proteobacteria (*Thiobacillus*, *Rhodococcus*, *Hyphomicrobium*) | (29) |

**References**

1. Howard EC, Henriksen JR, Buchan A, Reisch CR, Bürgmann H, Welsh R, Ye W, González JM, Mace K, Joye SB, Kiene RP, Whitman WB, Moran MA. 2006. Bacterial taxa that limit sulfur flux from the ocean. Science 314:649–652.

2. Reisch CR, Stoudemayer MJ, Varaljay VA, Amster IJ, Moran MA, Whitman WB. 2011. Novel pathway for assimilation of dimethylsulphoniopropionate widespread in marine bacteria. Nature 473:208–211.

3. Todd JD, Curson ARJ, Nikolaidou-Katsaraidou N, Brearley CA, Watmough NJ, Chan Y, Page PCB, Sun L, Johnston AWB. 2010. Molecular dissection of bacterial acrylate catabolism – unexpected links with dimethylsulfoniopropionate catabolism and dimethyl sulfide production. Environ Microbiol 12:327–343.

4. Curson ARJ, Rogers R, Todd JD, Brearley CA, Johnston AWB. 2008. Molecular genetic analysis of a dimethylsulfoniopropionate lyase that liberates the climate-changing gas dimethylsulfide in several marine α-proteobacteria and rhodobacter sphaeroides. Environ Microbiol 10:757–767.

5. Todd JD, Curson ARJ, Kirkwood M, Sullivan MJ, Green RT, Johnston AWB. 2011. DddQ, a novel, cupin-containing, dimethylsulfoniopropionate lyase in marine roseobacters and in uncultured marine bacteria. Environ Microbiol 13:427–438.

6. Todd JD, Kirkwood M, Newton-Payne S, Johnston AWB. 2012. DddW, a third DMSP lyase in a model Roseobacter marine bacterium, *Ruegeria pomeroyi* DSS-3. The ISME Journal 6:223–226.

7. Sun J, Todd JD, Thrash JC, Qian Y, Qian MC, Temperton B, Guo J, Fowler EK, Aldrich JT, Nicora CD, Lipton MS, Smith RD, De Leenheer P, Payne SH, Johnston AWB, Davie-Martin CL, Halsey KH, Giovannoni SJ. 2016. The abundant marine bacterium pelagibacter simultaneously catabolizes dimethylsulfoniopropionate to the gases dimethyl sulfide and methanethiol. Nat Microbiol 1:16065.

8. Curson ARJ, Sullivan MJ, Todd JD, Johnston AWB. 2011. DddY, a periplasmic dimethylsulfoniopropionate lyase found in taxonomically diverse species of proteobacteria. ISME J 5:1191–1200.

9. Wang S-Y, Zhang N, Teng Z-J, Wang X-D, Todd JD, Zhang Y-Z, Cao H-Y, Li C-Y. 2023. A new dimethylsulfoniopropionate lyase of the cupin superfamily in marine bacteria. Environ Microbiol 25:1238.

10. Todd JD, Curson ARJ, Dupont CL, Nicholson P, Johnston AWB. 2009. The *dddP* gene, encoding a novel enzyme that converts dimethylsulfoniopropionate into dimethyl sulfide, is widespread in ocean metagenomes and marine bacteria and also occurs in some ascomycete fungi. Environmental Microbiology 11:1376–1385.

11. Todd JD, Rogers R, Li YG, Wexler M, Bond PL, Sun L, Curson ARJ, Malin G, Steinke M, Johnston AWB. 2007. Structural and Regulatory Genes Required to Make the Gas Dimethyl Sulfide in Bacteria. Science 315:666–669.

12. Li C-Y, Wang X-J, Chen X-L, Sheng Q, Zhang S, Wang P, Quareshy M, Rihtman B, Shao X, Gao C, Li F, Li S, Zhang W, Zhang X-H, Yang G-P, Todd JD, Chen Y, Zhang Y-Z. 2021. A novel ATP dependent dimethylsulfoniopropionate lyase in bacteria that releases dimethyl sulfide and acryloyl-CoA. Elife 10:e64045.

13. Alcolombri U, Ben-Dor S, Feldmesser E, Levin Y, Tawfik DS, Vardi A. 2015. Identification of the algal dimethyl sulfide–releasing enzyme: a missing link in the marine sulfur cycle. Science 348:1466–1469.

14. Curson ARJ, Liu J, Bermejo Martínez A, Green RT, Chan Y, Carrión O, Williams BT, Zhang S-H, Yang G-P, Bulman Page PC, Zhang X-H, Todd JD. 2017. Dimethylsulfoniopropionate biosynthesis in marine bacteria and identification of the key gene in this process. Nat Microbiol 2:17009.

15. Williams BT, Cowles K, Bermejo Martínez A, Curson ARJ, Zheng Y, Liu J, Newton-Payne S, Hind AJ, Li C-Y, Rivera PPL, Carrión O, Liu J, Spurgin LG, Brearley CA, Mackenzie BW, Pinchbeck BJ, Peng M, Pratscher J, Zhang X-H, Zhang Y-Z, Murrell JC, Todd JD. 2019. Bacteria are important dimethylsulfoniopropionate producers in coastal sediments. Nat Microbiol 4:1815–1825.

16. Wang J, Curson ARJ, Zhou S, Carrión O, Liu J, Vieira AR, Walsham KS, Monaco S, Li C-Y, Dong Q-Y, Wang Y, Rivera PPL, Wang X-D, Zhang M, Hanwell L, Wallace M, Zhu X-Y, Leão PN, Lea-Smith DJ, Zhang Y-Z, Zhang X-H, Todd JD. 2024. Alternative dimethylsulfoniopropionate biosynthesis enzymes in diverse and abundant microorganisms. Nat Microbiol 9:1979–1992.

17. Trottmann F, Ishida K, Franke J, Stanišić A, Ishida-Ito M, Kries H, Pohnert G, Hertweck C. 2020. Sulfonium acids loaded onto an unusual thiotemplate assembly line construct the cyclopropanol warhead of a burkholderia virulence factor. Angew Chem (Int Ed, Engl) 59:13511–13515.

18. Curson ARJ, Williams BT, Pinchbeck BJ, Sims LP, Martínez AB, Rivera PPL, Kumaresan D, Mercadé E, Spurgin LG, Carrión O, Moxon S, Cattolico RA, Kuzhiumparambil U, Guagliardo P, Clode PL, Raina J-B, Todd JD. 2018. DSYB catalyses the key step of dimethylsulfoniopropionate biosynthesis in many phytoplankton. Nat Microbiol 3:430–439.

19. Kageyama H, Tanaka Y, Shibata A, Waditee-Sirisattha R, Takabe T. 2018. Dimethylsulfoniopropionate biosynthesis in a diatom *thalassiosira pseudonana*: identification of a gene encoding MTHB-methyltransferase. Arch Biochem Biophys 645:100–106.

20. Zhang Y, Sun C, Guo Z, Liu L, Zhang X, Sun K, Zheng Y, Gates AJ, Todd JD, Zhang X-H. 2024. An S-methyltransferase that produces the climate-active gas dimethylsulfide is widespread across diverse marine bacteria. Nat Microbiol 9:2614–2625.

21. Guo R, Guo Z, Zhou Y, Zhang Y, Cheng H, Devine R, Sun C, Liu R, Zheng Y, Gates AJ, Todd JD, Zhang X-H. 2026. Two novel *S* -methyltransferases confer dimethylsulfide production in *Actinomycetota*. Adv Sci 13:e10141.

22. McDevitt CA, Hugenholtz P, Hanson GR, McEwan AG. 2002. Molecular analysis of dimethyl sulphide dehydrogenase from rhodovulum sulfidophilum: its place in the dimethyl sulphoxide reductase family of microbial molybdopterin-containing enzymes. Mol Microbiol 44:1575–1587.

23. Horinouchi M, Kasuga K, Nojiri H, Yamane H, Omori T. 1997. Cloning and characterization of genes encoding an enzyme which oxidizes dimethyl sulfide in acinetobacter sp. strain 20B. FEMS Microbiol Lett 155:99–105.

24. Lidbury I, Kröber E, Zhang Z, Zhu Y, Murrell JC, Chen Y, Schäfer H. 2016. A mechanism for bacterial transformation of dimethylsulfide to dimethylsulfoxide: a missing link in the marine organic sulfur cycle. Environ Microbiol 18:2754–2766.

25. Bilous PT, Cole ST, Anderson WF, Weiner JH. 1988. Nucleotide sequence of the dmsABC operon encoding the anaerobic dimethylsulphoxide reductase of escherichia coli. Mol Microbiol 2:785–795.

26. Mouncey NJ, Choudhary M, Kaplan S. 1997. Characterization of genes encoding dimethyl sulfoxide reductase of rhodobacter sphaeroides 2.4.1T: an essential metabolic gene function encoded on chromosome II. J Bacteriol 179:7617–7624.

27. Boden R, Borodina E, Wood AP, Kelly DP, Murrell JC, Schäfer H. 2011. Purification and characterization of dimethylsulfide monooxygenase from hyphomicrobium sulfonivorans. J Bacteriol 193:1250–1258.

28. Eyice Ö, Myronova N, Pol A, Carrión O, Todd JD, Smith TJ, Gurman SJ, Cuthbertson A, Mazard S, Mennink-Kersten MASH, Bugg TDH, Andersson KK, Johnston AWB, Op Den Camp HJM, Schäfer H. 2018. Bacterial SBP56 identified as a Cu-dependent methanethiol oxidase widely distributed in the biosphere. ISME J 12:145–160.

29. Tanaka H, Esaki N, Soda K. 1977. Properties of L-methionine gamma-lyase from Pseudomonas ovalis. Biochemistry 16:100–106.
